# Prediction of heading date, culm length, and biomass from canopy-height-related parameters derived from time-series UAV observations of rice

**DOI:** 10.3389/fpls.2022.998803

**Published:** 2022-12-13

**Authors:** Shoji Taniguchi, Toshihiro Sakamoto, Ryoji Imase, Yasunori Nonoue, Hiroshi Tsunematsu, Akitoshi Goto, Kei Matsushita, Sinnosuke Ohmori, Hideo Maeda, Yoshinobu Takeuchi, Takuro Ishii, Jun-ichi Yonemaru, Daisuke Ogawa

**Affiliations:** ^1^ Research Center for Agricultural Information Technology, National Agricultural and Food Research Organization (NARO), Tsukuba, Japan; ^2^ Institute of Crop Science, National Agricultural and Food Research Organization (NARO), Tsukuba, Japan; ^3^ Institute for Agro-Environmental Sciences, National Agricultural and Food Research Organization (NARO), Tsukuba, Japan

**Keywords:** UAV, canopy height, rice, heading date, biomass, time-series, BLUP

## Abstract

Unmanned aerial vehicles (UAVs) are powerful tools for monitoring crops for high-throughput phenotyping. Time-series aerial photography of fields can record the whole process of crop growth. Canopy height (CH), which is vertical plant growth, has been used as an indicator for the evaluation of lodging tolerance and the prediction of biomass and yield. However, there have been few attempts to use UAV-derived time-series CH data for field testing of crop lines. Here we provide a novel framework for trait prediction using CH data in rice. We generated UAV-based digital surface models of crops to extract CH data of 30 Japanese rice cultivars in 2019, 2020, and 2021. CH-related parameters were calculated in a non-linear time-series model as an S-shaped plant growth curve. The maximum saturation CH value was the most important predictor for culm length. The time point at the maximum CH contributed to the prediction of days to heading, and was able to predict stem and leaf weight and aboveground weight, possibly reflecting the association of biomass with duration of vegetative growth. These results indicate that the CH-related parameters acquired by UAV can be useful as predictors of traits typically measured by hand.

## Introduction

Phenotyping is a fundamental procedure in field testing of crops and is typically done laboriously by hand. To make phenotyping more effective, various methods using unmanned aerial vehicles (UAVs) have been developed for measuring crop physical parameters, especially in the field (Furbank and Tester, 2011; Ninomiya, 2022). UAVs can carry several types of cameras, including RGB (red–green–blue), multispectral, and thermal infrared, to take images of crops (Yang et al., 2017; Sakamoto et al., 2022). From RGB images, the 2D vegetation fraction and vertical canopy height (CH) can be extracted (Ogawa et al., 2021a; Ogawa et al., 2021b). Vegetation indices, obtained by spectral analysis, such as the Normalized Difference Vegetation Index (NDVI), have been used for estimating nitrogen use efficiency (Liang et al., 2021), drought resistance (Jiang et al., 2021), and lodging (Yadav et al., 2017; Singh et al., 2019), and for predicting biomass and yield (Yue et al., 2017; Gong et al., 2018; Di Gennaro et al., 2019; Duan et al., 2019; Wang et al., 2019a). These attempts indicate the usefulness of UAVs for high-throughput phenotyping of crops in the field.

Rice is a staple food, especially in Asia (Muthayya et al., 2014). Crucial to increased and sustainable rice production, yield and biomass are complex traits affected by plant shape and size (Peng et al., 2008; Xing and Zhang, 2010; Ikeda et al., 2013). Culm length (CL), panicle length (PL), and panicle number (PN), values of which reflect the genetic architecture of rice, are roughly related to yield and biomass (Zhao et al., 2011). Breeding for longer culms led to the selection of a rice line with higher grain yield and plant weight (Nomura et al., 2019). A rice line carrying *OsglHAT1*, which encodes a new-type GNAT-like protein that harbors intrinsic histone acetyltransferase activity, had increased plant size and grain length and width, with increased yield and biomass (Song et al., 2015). Panicle length (PL) and panicle number (PN) are strongly related to rice yield (Agata et al., 2020; Liu et al., 2022). Growth period also influences rice yield and biomass (Endo-Higashi and Izawa, 2011; Gao et al., 2014), and days to heading (DTH) is generally used to evaluate the transition from vegetative to reproductive stage. Conventionally, CL, PL, PN, and DTH are measured by hand at high cost. For rice breeding and examining the cultivation competence of cultivars at lower cost, a practical high-throughput phenotyping system to estimate these traits in the field is required.

In our previous study, rice CH estimated from UAV images was highly correlated with CL (Ogawa et al., 2021b), making it a potential predictor of yield and biomass. A promising approach to make the most of UAV-based CH data for rice phenotyping is time-series monitoring, in which remote sensing has an advantage by being non-invasive and non-destructive. Estimation of plant height in maize inbred lines at four growth stages by UAV showed that temperate lines grew faster at early growth stages, but tropical lines grew faster at later growth stages (Wang et al., 2019b). Time-series observations of rice CH with UAV correlated highly with CH measured by hand, and revealed growth patterns and differences in functional stages of quantitative trait loci for CH (Ogawa et al., 2021b). Use of a cable-suspended phenotyping platform allowed the temperature response of CH in wheat lines to be clarified (Kronenberg et al., 2021). These studies revealed time-series CH dynamism as a new feature different from one-off CH measurement and led to the hypothesis that time-series CH analysis could reveal genetic and phenological characteristics of rice cultivars and predict yield-related traits usually measured by hand.

One of the important challenges in time-series data analysis is handling time-series changes to allow comparison (Giorgino, 2009; Sugihara et al., 2012; Maziarz, 2015). Many time-series models have been proposed for analyzing crop phenology. Such models include shape-model fitting (Sakamoto et al., 2013; Zhou et al., 2020), random regression with the Legendre polynomial (Campbell et al., 2018; Campbell et al., 2019), segmented linear regression (Toda et al., 2021), and non-linear growth curves (Chang et al., 2017; Grados et al., 2020; Poudel et al., 2022). Anderson et al. (2019) applied a three-parameter logistic model (S-shape non-linear curve) to maize CH time-series data measured by UAV over 1 year, applied a linear mixed effects (LME) model to the logistic parameters, decomposed the parameter variance into genetic and environmental effects: they showed that some of the parameters could be used as predictors of grain yield. Borra-Serrano et al. (2020) and Chang et al. (2017) applied similar S-shape non-linear curves to, respectively, soybean and sorghum CH time-series data measured by UAV. In contrast to these crops, in which CH increases with plant growth, rice CH decreases in the reproductive stage. Therefore, it is necessary to develop a new model to incorporate the effects of the CH decrease and its timing, and to apply it to CH time-series data covering various rice lines.

In this study, we aimed at revealing how UAV-derived time-series CH data are useful for predicting yield and biomass and related traits such as DTH, CL, PL and PN. We developed a novel time-series model incorporating both CH increase and decrease during the growth period, unlike previous models developed for maize, soybean, and sorghum. To develop our model, we used data covering 3 years and 30 cultivars, enabling us to evaluate its robustness and to analyze the cultivar effects by LME models. Through this analysis, we developed a practical and high-throughput method for the prediction of rice traits from CH-related parameters.

## Materials and methods

### Growing of rice cultivars

**Figure 1:**
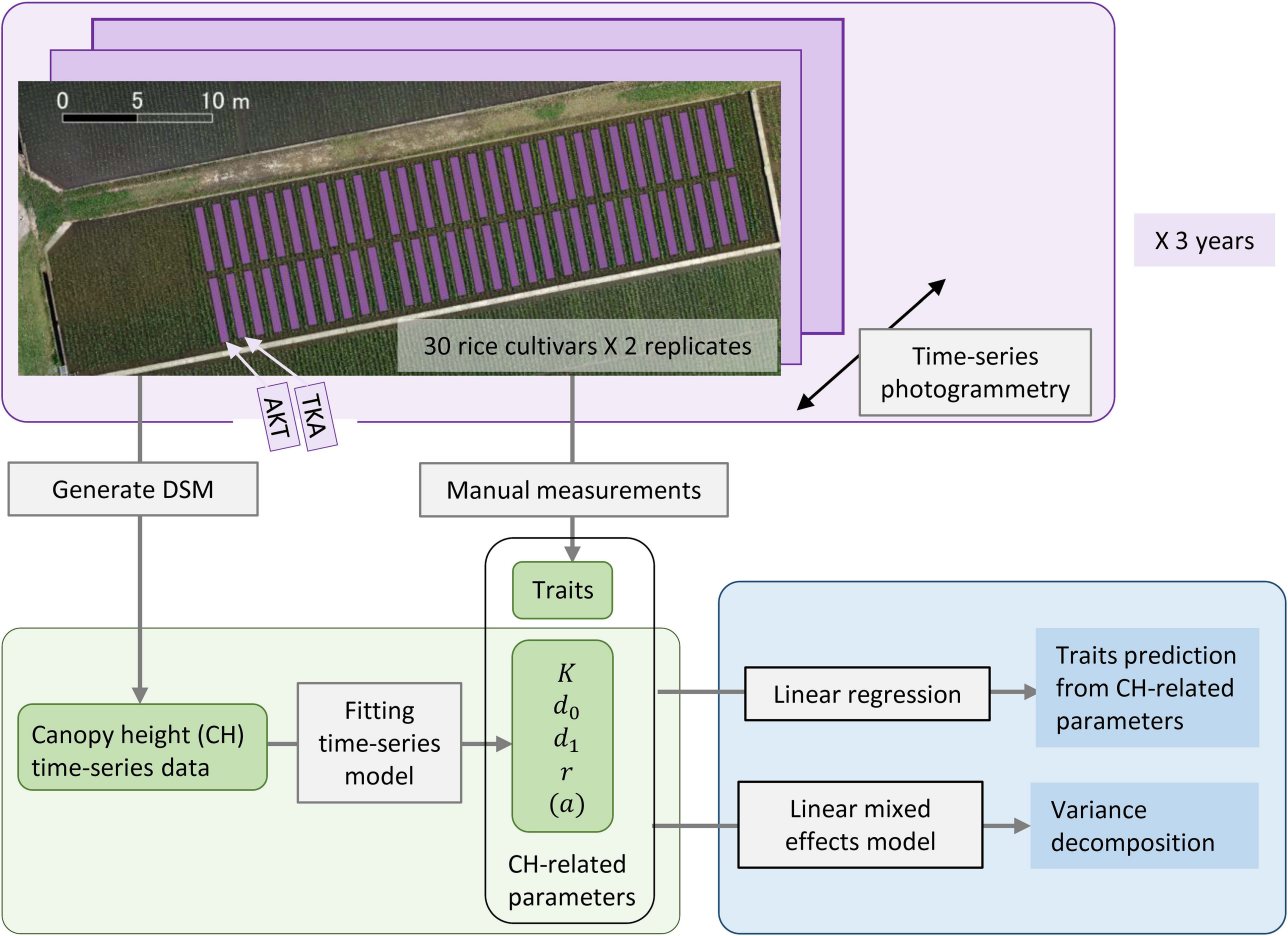
Framework of our analysis for the traits prediction from CH-related parameters. Thirty rice cultivars including AKT and TKA were grown with 2 replicates in 2019, 2020, and 2021. Aerial photogrammetry was conducted weekly. CH time-series data were obtained by generating a DSM and CH-related parameters were calculated. Several traits were measured by hand. Statistical analysis included linear regression for the traits prediction and linear mixed effects modeling for the variance decomposition of the traits and CH-related parameters.

Seeds of 30 rice cultivars in Japan, including those developed for high grain yield, lodging resistance, disease resistance, and brown rice quality (**Supplementary Table 1**), were sown in seedling medium on 17 April 2019, 20 April 2020, and 20 April 2021. We transplanted 3 seedlings per hill at a density of 22.2 plants/m^2^ into a paddy field in Tsukubamirai city (36°00′33″N, 140°01′20″E), Japan, on 17 May 2019, 15 May 2020, and 13 May 2021. The paddy field was divided into 60 plots, two per cultivar (**Figure 1**). The size of each plot was 2.7 m^2^. The plants were grown in the field for about 5 months.

### UAV-based aerial photography

Aerial observations were made about once a week as in our previous studies (Ogawa et al., 2019; Ogawa et al., 2021a; Ogawa et al., 2021b). We used a Phantom 4 Pro UAV (P4P; DJI, Shenzhen, China) to capture RGB images with an onboard 20-megapixel camera that flew automatically at 1.0 m/s over the paddy field at an altitude of 10.3 m. DJI GS Pro software controlled the flight path and set the following photogrammetry conditions: capture mode, time interval; front overlap ratio, 80%; side overlap ratio, 80%; gimbal pitch angle, −90°, white balance, cloudy; aperture, auto; shutter, auto. Each flight took 150–200 images covering the field, each measuring 5472 × 3648 pixels. To set the focus, the P4P was manually raised to 10.3 m, the camera was focused automatically on a region of the canopy, and then the focus mode was changed to manual. We placed seven ground control point (GCP) markers on the ground around the test field. We obtained the altitude, longitude, and latitude of each GCP by real-time kinematic positioning using a DG-PRO1RWS receiver (BizStation Corp., Matsumoto, Japan).

### Generation of digital surface model and quantification of CH

As previously, Agisoft MetaShape Professional v. 1.6.5 software (Agisoft, St. Petersburg, Russia) generated a digital surface model (DSM) from each image set (150–200 images per set) by the date of photogrammetry in the following procedure: (1) align photos (high accuracy), (2) import GCPs, (3) optimize camera, (4) build dense cloud, (5) build digital elevation model (source data to be dense cloud), and (6) export the digital elevation model. The coordinate system was set to UTM zone 54N (WGS-84) and the resolution to 2 mm/pixel. Next, QGIS (3.20.0) software (QGIS Development Team) cut out the area of the paddy field from each DSM image and identified the position of each plot to create shape files. Finally, a script written in Python (3.9.7: Python Software Foundation) cut out the portion in the DSM images corresponding to each plot in reference to the shape files. The computer was an AMD Ryzen Threadripper 2990WX (32-Core Processor, 3.00 GHz, 128 GB RAM, GeForce RTX 2080 Ti GPU) running the 64-bit Windows 10 Pro operating system.

We defined the canopy position as the 95th percentile in the cut-out DSMs corresponding to each plot. CH was defined as the difference between canopy position and ground level. We defined ground level as the 2nd percentile just after transplanting in the cut-out DSMs corresponding to each plot.

### Fitting time-series model to the CH data

**Figure 2:**
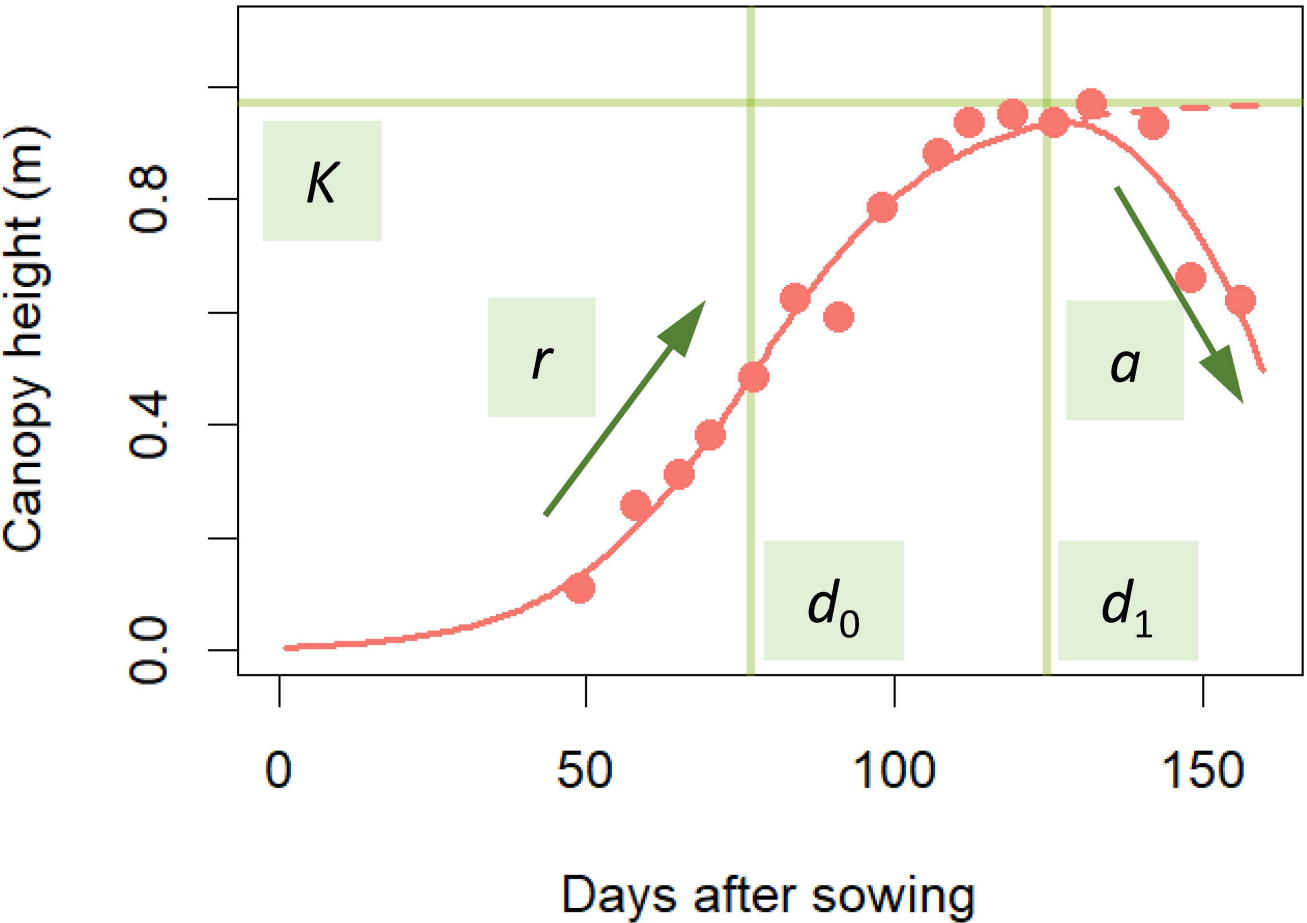
Fitting time-series model to CH time-series data. Red points are CH data obtained by UAV. The time-series model applied to the data is the red S-shaped curve. The model was prescribed by 5 parameters: *K*, the maximum saturation value; *r*, growth rate; *a*, CH decrease in the late growth phase; *d*_0_, time point at the highest growth rate; *d*_1_, time point at maximum CH. This curve was described in equation (1).

For the statistical modeling of the CH time-series data, we adopted a three-parameter logistic as the typical model for the S-shape plant growth (Paine et al., 2012). Since the logistic asymptotically approaches the maximum saturation value *K*, we modified it to incorporate the CH decrease in the late growth phase to develop the following time-series model:


(1) $$\begin{array}{c} CH=\{\begin{array}{c} \frac{K}{1+\text{exp}\left(r\left(d_{0}-x\right)\right)}\;\left(when\;x\leq d_{1}\right),\\ \frac{K}{1+\text{exp}\left(r\left(d_{0}-x\right)\right)}-a{\left(x-d_{1}\right)}^{2}\;(when\;x>d_{1}), \end{array} \end{array}$$

where *x* is days after sowing, *d*_0_ is the time point at the highest growth rate, *d*_1_ is the time point at the maximum CH, *r* is the growth rate, and *a* is the CH decrease rate from the logistic S-shape curve in the late growth phase (**Figure 2**).

For parameter estimation, we used a two-step procedure to prevent false convergence in the estimation algorithm. First, we calculated *K* and *d*_1_, taking the maximum value in each CH time-series data set as *K*. We fitted a cubic polynomial to each data set and obtained *d*_1_ as the time point when the cubic polynomial was at its maximum. We applied the following cubic polynomial:


(2) $$\begin{array}{c} CH=\beta_{0}+\beta_{1}x+\beta_{2}x^{2}+\beta_{3}x^{3}. \end{array}$$

Second, we fitted equation 1 given the values of *K* and *d*_1_. Except for *K*, all parameters were obtained by means of the nonlinear least squares method implemented in R (R Core Team, 2021). For parameter estimation for equation 1, we used the R function *nls*, adopting the nl2sol algorithm and setting the initial values to *d*_0_=50 , *r*=0.05 , and *a*=1.0 × 10^−4^ .

### Manual measurement of traits related to yield

Heading date was defined as the date when panicles emerged from about half of the stems in each plot. DTH (days) was the period from the sowing date to the heading date. CL (m) and PL (cm) of the longest culm of each plant were measured and PN was counted once from 2 to 4 weeks after heading. CL was defined as the length from the ground to the panicle base, and PL was defined as the length from there to the tip of the spikelet. Mean values from 10 plants per cultivar were used for CL, PL, and PN. For aboveground dry weight (ADW; g), 50 plants per plot at maturity were harvested from the ground and air-dried for more than 2 weeks before the measurement. Stem and leaf weight (SLW; g) was obtained as ADW − grain weight (GW; g). These traits were measured in each plot. All trait names are listed in **Table 1**.

**Table 1:** Traits and their abbreviations.

Abbreviation	Trait
ADW	Aboveground dry weight
CL	Culm length
DTH	Days to heading
GW	Grain weight
PL	Panicle length
PN	Panicle number
SLW	Stem and leaf weight

### Statistical analysis of traits and parameters

To decompose the traits and CH-related parameters into cultivar, year, and cultivar × year interaction effects, we applied the linear mixed effects (LME) model:


(3) $$\begin{array}{c} X_{lyb}=\mu_{X}+X_{l}+X_{y}+X_{ly}+\epsilon , \end{array}$$

where *X_lyb_* is a parameter or trait of cultivar *l* in year *y* and plot *b*; µ*_X_* is the fixed effect for the average value; $X_{l}\sim N\left(0,\sigma_{l}^{2}\right)$ , $X_{y}\sim N\left(0,\sigma_{y}^{2}\right)$ , and $X_{ly}\sim N\left(0,\sigma_{ly}^{2}\right)$ are random effects of cultivar, year, and cultivar × year interaction, respectively; and $\epsilon \sim N\left(0,\sigma_{\epsilon }^{2}\right)$ is the residual. We defined heritability as the ratio of cultivar variance to the total variance:


(4) $$\begin{array}{c} h^{2}=\frac{\sigma_{l}^{2}}{\sigma_{l}^{2}+\sigma_{y}^{2}+\sigma_{ly}^{2}+\sigma_{\epsilon }^{2}}. \end{array}$$

The R package *lme4* (Bates et al., 2015) estimated the parameters and the best linear unbiased predictors (BLUPs) of the LME model by the REML method. Total variance was calculated as follows:


$$\sigma_{All}^{2}=\sigma_{l}^{2}+\sigma_{y}^{2}+\sigma_{ly}^{2}+\sigma_{\epsilon }^{2}$$

**Figure 3:**
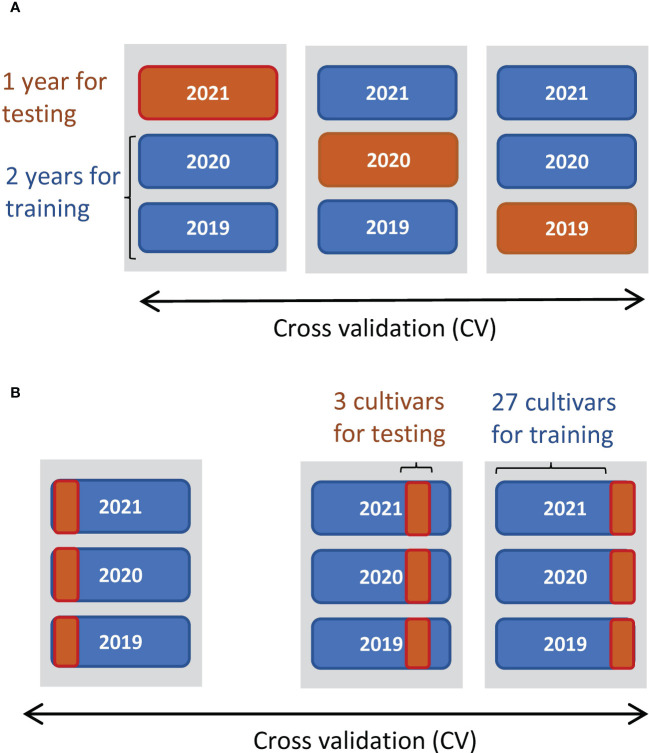
Schemes of CV to predict manually measured traits. **(A)** Threefold CV, where data were split by year: two years were used for training data and the other as test data. **(B)** Tenfold CV, where data were split by cultivar: 27 cultivars as training data and the other 3 as test data.

We used the linear regression model to predict the yields from the CH-related parameters and evaluated whether the phenology data contained enough information about yield. To reveal what CH-related parameters are useful for the prediction of traits, we examined Pearson’s correlations (cor). Since multicollinearity impairs the accuracy of regression coefficients, we used backward variable selection to prevent it. We calculated the variance inflation factor in the R package *car* (Fox and Weisberg, 2019) for variable selection and adopted four parameters as predictors without multicollinearity (variance inflation factor< 5): *K*, *d*_0_, *d*_1_, and *r* (**Supplementary Table 2**). All four predictors were standardized to have a mean of 0 and standard deviation of 1. Next, we constructed linear regression models by the ordinary least squares method to predict CL, DTH, ADW, GW, and SLW. The prediction accuracies were evaluated by cross-validation (CV), splitting data by year and by cultivar (**Figure 3**). Finally, the regression coefficients were estimated from all data (*n* = 180). As measures of accuracy, we used cor and root-mean-square error (RMSE) between observed and predicted values of test data. RMSE evaluates the accuracy of predicting the exact values, and cor evaluates the accuracy of predicting the magnitude of correlation.

### Influence of accumulated daily mean temperature on CH-related parameters *d*_0_ and *d*_1_

We transformed *d*_0_ and *d*_1_ values to accumulated temperature *d*_0_ *^temp^* and *d*_1_ *^temp^*, starting from the planting date to the date of *d*_0_ or *d*_1_:


$$d_{0}^{temp}=\sum_{d=d_{p}+1}^{d_{0}}T_{d}$$

$$d_{1}^{temp}=\sum_{d=d_{p}+1}^{d_{1}}T_{d}$$

where *T_d_* is the mean temperature of day *d* and *d*_p_ is the date of planting. The base temperature was set to 0°C. We used the LME model in equation 2. Daily mean temperature (°C) in the paddy field is shown in **Supplementary Data 1**-**3**.

## Results

### Relationships between manually measured traits and CH-related parameters in 30 rice cultivars

We characterized phenotype data of 30 Japanese rice cultivars (**Supplementary Table 1**) in 2019, 2020, and 2021 from the aspect of genetics and examined how to use the CH data for the prediction of traits usually measured by hand (**Figure 1**).

**Figure 4:**
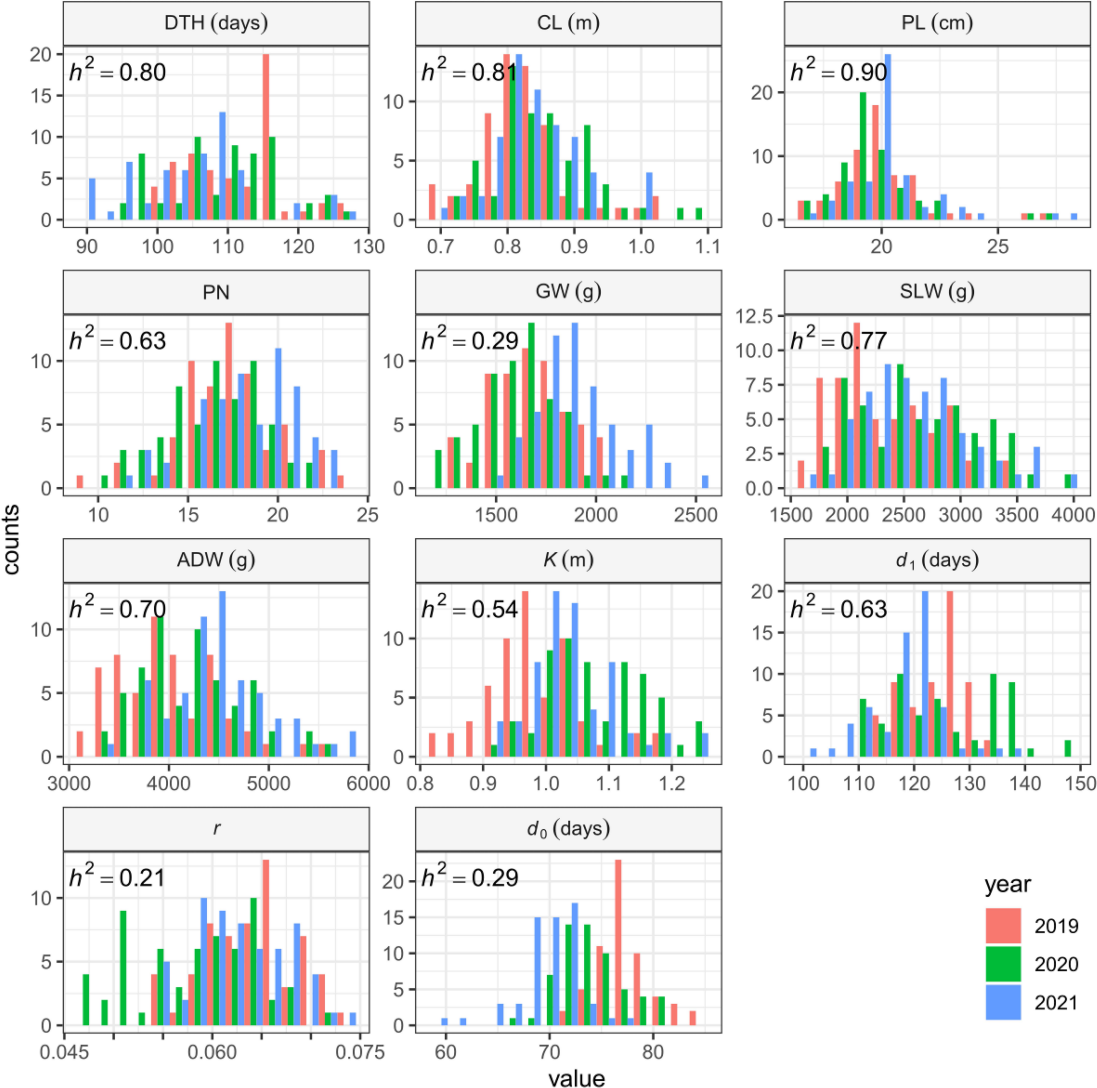
Frequency distributions of manually measured traits and CH-related parameters, shown as histograms.

The sizes of interannual differences in phenotypic distribution depended on trait (**Figure 4**; **Table 2**; **Supplementary Table 3**). Distributions of DTH, CL, PL, and PN were highly overlapped among years, and heritability was high: that of DTH was 0.80, CL 0.81, PL 0.90, and PN 0.63 (**Table 2**). On the other hand, the phenotypic distribution of GW was wider than that of SLW, especially between 2021 and the other 2 years (**Figure 4**). Consistent with this, heritability of GW was 0.29, much lower than that of SLW at 0.77. Therefore, GW was more susceptible to year effect than SLW (**Table 2**). ADW is SLW + GW, and its heritability (0.70) was positioned between theirs.

**Table 2:** Summary statistics of CH-related parameters and traits obtained by the linear mixed effects model.

Trait	Heritability	Mean	σ_All_^2^ ^a^	σ_l_^2^ ^b^	σ_y_^2^ ^c^	σ_ly_^2^ ^d^	σ_ϵ_^2^ ^e^
DTH	0.80	108.6	7.3 × 10	5.9 × 10	1.2 × 10	1.8	2.6 × 10^−1^
CL	0.81	840.3 × 10^−3^	5.2 × 10^−3^	4.3 × 10^−3^	2.0 × 10^−4^	1.8 × 10^−4^	5.9 × 10^−4^
PL	0.90	200.4 × 10^−1^	3.7	3.4	1.0 × 10^−1^	5.1 × 10^−2^	2.2 × 10^−1^
PN ^f^	0.63	173.2 × 10^−1^	8.2	5.2	6.5 × 10^−1^	0.0	2.4
GW	0.29	173.0 × 10	6.5 × 10^4^	1.9 × 10^4^	2.3 × 10^4^	1.2 × 10^4^	1.2 × 10^4^
SLW	0.77	252.4 × 10	2.7 × 10^5^	2.1 × 10^5^	2.3 × 10^4^	1.3 × 10^4^	2.7 × 10^4^
ADW	0.70	425.4 × 10	3.3 × 10^5^	2.3 × 10^5^	5.2 × 10^4^	5.5 × 10^3^	4.2 × 10^4^
*K*	0.54	103.5 × 10^−2^	8.0 ×10^−3^	4.3 ×10^−3^	2.3 ×10^−3^	8.1 ×10^−4^	6.1 ×10^−4^
*d*_1_	0.63	122.8	7.6 × 10	4.8 × 10	1.5 × 10	1.2 × 10	7.4 × 10^−1^
*r*	0.21	617.2 × 10^−4^	3.7 × 10^−5^	7.7 × 10^−6^	8.9 × 10^−6^	1.8 × 10^−5^	2.4 × 10^−6^
*d*_0_	0.29	737.3 × 10^−1^	2.3 × 10	6.8	1.4 × 10	1.7	4.8 × 10^−1^
*a*	0.61	385.6 × 10^−6^	3.1 ×10^−8^	1.9 ×10^−8^	1.4 ×10^−9^	9.7 ×10^−9^	1.1 ×10^−9^

a Total variance.

b Variance of cultivars.

c Variance of years.

d Variance of cultivar × year interaction.

e Variance of residuals.

f Note that the result of PN was singular.

We collected 2834 CH data of the 30 Japanese cultivars over the 3 years (**Supplementary Table 4**). Given the huge size of the dataset, we obtained CH-related parameters by applying time-series curves to the CH data by plot (**Supplementary Figure 1**). The range of phenotypic distributions tended to differ by year (**Figure 4**; **Supplementary Table 3**). Heritabilities of *K* (0.54) and *d*_1_ (0.63) were higher than those of *r* (0.21) and *d*_0_ (0.29), suggesting that *r* and *d*_0_, parameters of vegetative growth, might also be more susceptible to year effects than *K* and *d*_1_, parameters of the reproductive stage (**Table 2**).

**Figure 5:**
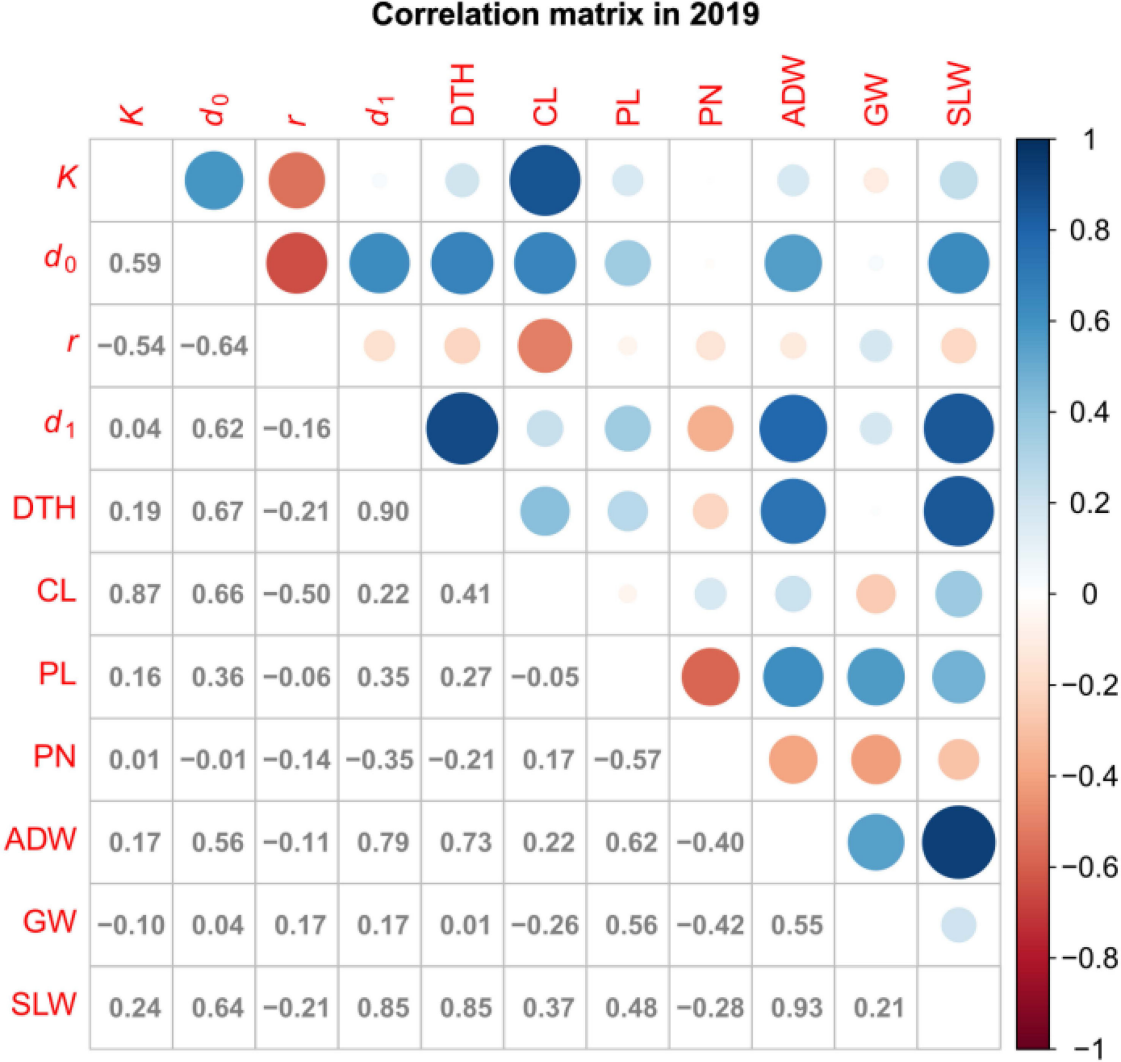
Correlation plot of parameters and traits in 2019. Values are correlation coefficients (cor); circles present them by color and size.

We obtained correlation plots between the parameters and traits (**Figure 5**; **Supplementary Figure 2**). *K* was positively correlated with CL. This result is consistent with our previous data showing high correlation between CH and CL in several rice lines (Ogawa et al., 2021b). *d*_1_ was positively correlated with DTH, ADW, and SLW. These results motivated us to use *K* and *d*_1_ to predict traits.

### Prediction of CL and DTH from CH-related parameters

**Figure 6:**
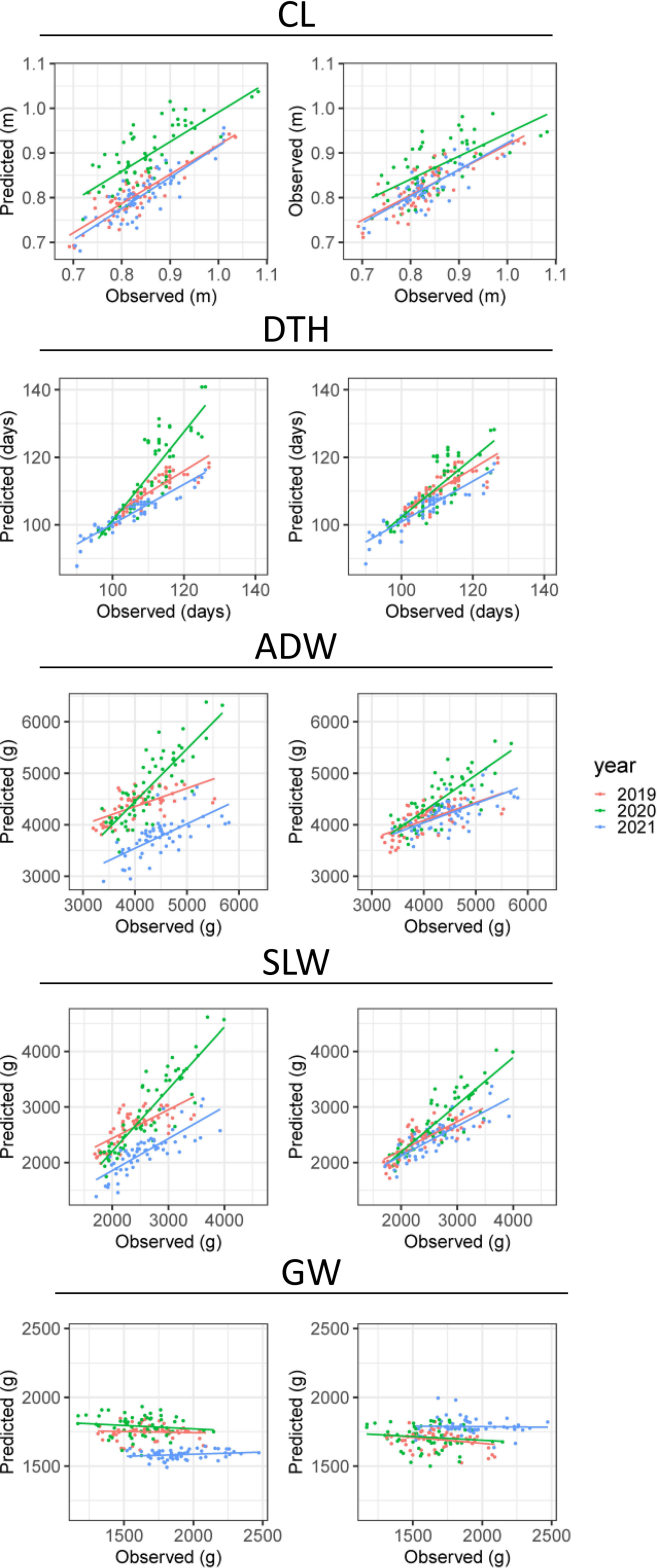
Observed and predicted values of CL, DTH, ADW, SLW, and GW. Plots show the results of CV by (left) year and (right) cultivar. The points plot datasets of predicted and observed values, colored by year; the lines are linear regressions applied to them.

CV indicated the accuracy of predicting CL and DTH from CH-related parameters (**Table 3**). In predicting the magnitudes of CL, cor_year_ = 0.82 and cor_cultivar_ = 0.68; and of DTH, cor_year_ = 0.89 and cor_cultivar_ = 0.85. The scatter plots between observed and predicted CL and DTH were highly correlated (**Figure 6**). In predicting the exact values of CL, RMSE_year_ = 0.05 m and RMSE_cultivar_ = 0.04 m; and of DTH, RMSE_year_ = 5.2 days and RMSE_cultivar_ = 4.2 days (**Table 3**). These RMSE values were smaller than the total standard deviations, the square root of the total variances (**Table 2**). These results indicate that the CH-related parameters had information that could be used to predict CL and DTH.

**Table 3:** Prediction accuracy of five traits evaluated by CV by year and by cultivar.

CV by year			CV by cultivar	
	cor_year_	RMSE_year_	cor_cultivar_	RMSE_cultivar_
CL	0.823	522.9 × 10^−4^	0.682	442.3 × 10^−4^
DTH	0.890	515.1 × 10^−2^	0.851	420.6 × 10^−2^
ADW	0.716	638.6	0.620	406.7
GW	0.003	294.6	0.312	232.6
SLW	0.808	437.0	0.743	291.9

cor, Pearson’s correlation coefficient; RMSE, root mean square error.

We calculated the regression coefficients in the regression models. Since all predictors were standardized, the importance of each parameter in the model was quantified as the absolute value of each coefficient. The regression model to predict CL was


(5) $$\begin{array}{c} \begin{array}{c} CL=0.84+0.06K+0.01d_{0}+0.01d_{1}+0.01r \end{array} \end{array}$$

and the coefficient of determination was *R* ^2^=0.684. The model to predict DTH was


(6) $$\begin{array}{c} \begin{array}{c} DTH=108.6+0.1K+1.3d_{0}+6.8d_{1}+1.1r \end{array} \end{array}$$

and $R^{2}=0.794$

In predicting CL, the coefficient of *K* (0.06, significant by *t*-test at 0.1%; **Supplementary Table 5**) had the largest absolute value, more than 4× the second largest one, that of *d*_0_ (0.01). In predicting DTH, the coefficient of *d*_1_ (6.82, significant by *t*-test at 0.1%; **Supplementary Table 5**) had the largest absolute value, more than 5× the second largest one, that of *d*_0_ (1.34). Therefore, in the linear regression model, *K* was the most important predictor of CL, and *d*_1_ was the most important predictor of DTH.

**Figure 7:**
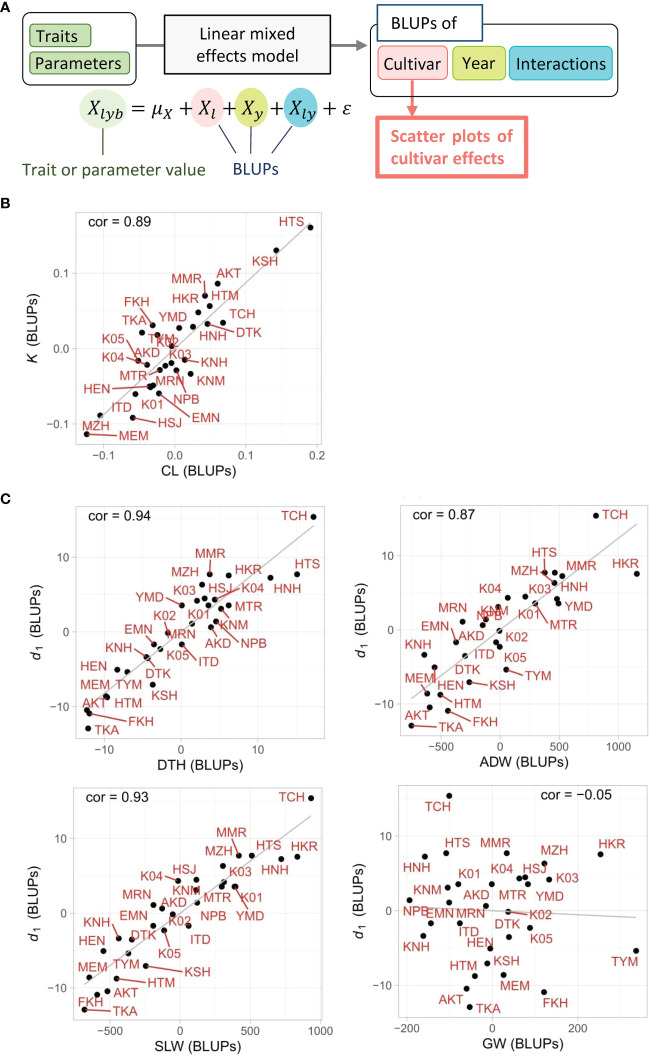
Scatter plots of cultivar effects between CH-related parameters and manually measured traits. **(A)** Flow chart of the process to generate scatter plots. The cultivar effect (*X_l_*) on traits and parameters was extracted from the linear mixed effects model as BLUPs from the 3-year experiment. **(B, C)** Plots of cultivar effects showing correlations between **(B)** *K* and CL and between **(C)** *d*_1_ and DTH, ADW, SLW, or GW. Correlation coefficients (cor) are shown in plots. Cultivar name codes are shown in red.

The linear regression models based on CH-related parameters explained the total variances of the manually measured traits, but it was still uncertain whether the relations between the two were derived from the characteristics of each cultivar. The LME model, which decomposed the total variance into cultivar, year, cultivar × year interactions, and residual, extracted the cultivar effects as the BLUPs from the whole data. First, the high heritabilities of CL (0.81) and DTH (0.80) imply that a large proportion of the total variance derived from cultivar effects. Note that heritability is the ratio of cultivar effect to the total variance. Second, the cultivar effects of CL, DTH, *K*, and *d*_1_, quantified as the BLUPs of each cultivar, showed a clear tendency that the cultivars with smaller *K* had a smaller CL, and those with smaller *d*_1_ had a smaller DTH (**Figure 7**). The correlation of cultivar BLUPs between *K* and CL was cor = 0.89, and that of *d*_1_ and DTH was cor = 0.94. These results indicate that the total variances of CL and DTH were largely prescribed by the cultivar effects of *K* and *d*_1_, respectively.

### Prediction of ADW, GW, and SLW from CH-related parameters

As it did for DTH and CL, our CV method gave the accuracy of prediction of ADW, GW, and SLW (**Table 3**). In predicting the magnitudes of ADW, cor_year_ = 0.72 and cor_cultivar_ = 0.62 and of SLW, cor_year_ = 0.81 and cor_cultivar_ = 0.74 (**Figure 6**). These values were better than predicting the magnitude of GW: cor_year_ = 0.00 and cor_cultivar_ = 0.31. The scatter plots between observed and predicted SLW and ADW were highly correlated. In predicting the exact values of ADW, cross-validation by year (RMSE_year_ = 406.7 g) had better accuracy than that by cultivar (RMSE_cultivar_ = 638.6 g), as had that of SLW (**Table 3**). The CH-related parameters contained information with which to predict ADW and SLW, but yearly fluctuations could increase RMSE. By contrast, as shown in the scatter plot between observed and predicted GW (**Figure 6**), the slopes were almost flat and the model explained little of the GW variance. Therefore, CH-related parameters held little information with which to predict GW, at least under our linear regression model.

The regression coefficients indicated the importance of each parameter in our regression models. (All predictors were standardized.) The regression model to predict ADW was


(7) $$\begin{array}{c} \begin{array}{c} ADW=4254+176K-242d_{0}+518d_{1}+132r \end{array} \end{array}$$

and *R* ^2^=0.475 . The model to predict SLW was


(8) $$\begin{array}{c} \begin{array}{c} SLW=2524+165K-199d_{0}+541d_{1}+105r \end{array} \end{array}$$

and *R* ^2^=0.684 . In predicting ADW and SLW, all four parameters were significant by *t*-test at 0.1%, and *d*_1_ had the largest absolute values (**Supplementary Table 5**). For ADW, the coefficient of *d*_1_ was 518, more than 2× the absolute value of *d*_0_ (−242), the second largest. Similarly, for SLW, the coefficient of *d*_1_ was 540, more than 2× the absolute value of *d*_0_ (−198). Therefore, *d*_1_ was the most important predictor of ADW and SLW.

The results of the LME model uncovered the effect of each cultivar on the total variance of ADW and SLW. The high heritabilities of ADW (0.70) and SLW (0.77) imply that a large proportion of total variance derived from cultivar effects. The cultivar effects of ADW, SLW, and *d*_1_, quantified as the BLUPs of each cultivar, showed a clear tendency in which cultivars with smaller *d*_1_ had smaller ADW and SLW (**Figure 7C**). On the other hand, the cultivar BLUPs of GW had little relation with those of *d*_1_. The correlations of cultivar BLUPs of *d*_1_ with SLW (cor = 0.93) and ADW (cor = 0.87) were higher than that with GW (cor = −0.05). The cultivar effects of *d*_1_ clearly reflected those of SLW. As ADW = SLW + GW, since the low correlation indicates that the cultivar effects of *d*_1_ and GW were almost independent, the result that the cultivar effects of *d*_1_ corresponded to those of ADW derives from the relation of the cultivar effects of *d*_1_ and SLW (**Figure 7C**; **Supplementary Figures 3**-**5**).

### Sensitivity of accumulated daily mean temperature to CH data

**Figure 8:**
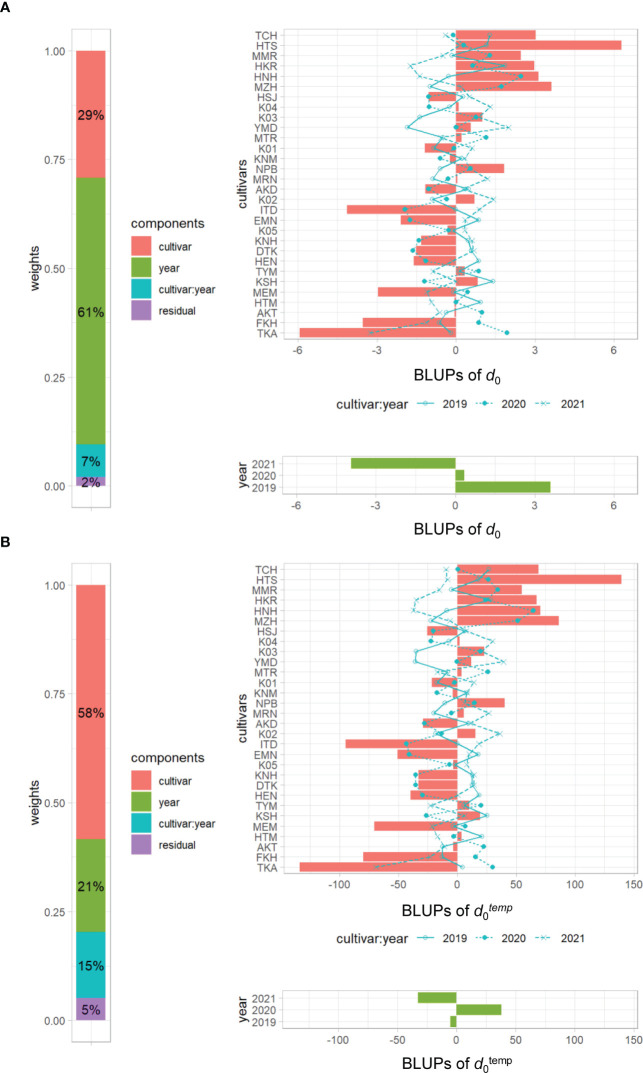
Visualization of the linear mixed effects model for **(A)** *d*_0_ and **(B)** *d*_0_ *^temp^*. Left, proportions of 4 variance components; right top, BLUPs of cultivar and cultivar × year interactions of 30 cultivars; right bottom, BLUPs of 3 years. Cultivars are sorted by *d*_1_. The numbers in cultivar components indicate percentage heritability.

The frequency distributions of the CH-related parameters differed among years (**Figure 4**), indicating that those may be influenced by environmental factors. In the developmental rate model, which is well known for the prediction of DTH in rice, daylength and daily mean temperature are explanatory variables (Horie et al., 1995). We asked whether the change of CH is affected by accumulated daily mean temperature instead of daylength, because the former varied among years (**Supplementary Data 1**-**3**), whereas daylength was almost constant owing to the similar planting dates. CH-related parameters *d*_0_ and *d*_1_ are based on time-series data, but *d*_0_ *^temp^* and *d*_1_ *^temp^* are based on accumulated daily mean temperature. Transforming *d*_0_ into *d*_0_ *^temp^* increased heritability from 0.29 to 0.58 (**Supplementary Table 6**) and decreased the year effect from 61% to 21% (**Figure 8**), meaning that the year effect on *d*_0_ was explained mostly by the accumulated temperature. On the other hand, the heritability of *d*_1_ *^temp^* (0.62) was almost the same as that of *d*_1_ (**Supplementary Table 6**; **Supplementary Figure 6**). These results indicate that the time point at the maximum CH (*d*_1_), which is linked to heading date, is insensitive to accumulated daily mean temperature, but that at the highest CH growth rate (*d*_0_) is sensitive to it.

## Discussion

We constructed a time-series model and applied it to the data of 30 rice cultivars in 2019, 2020, and 2021, which were derived from UAV-based time-series aerial photography. In the case of maize (Anderson et al., 2019), soybean (Borra-Serrano et al., 2020) and sorghum (Chang et al., 2017), CH continues to increase, and there is little need to consider the difference of CH growth in between vegetative and reproductive stages. On the other hand, the CH decrease in the reproductive stage is distinct in rice. In our model, we introduced the parameters *d*_1_, the time point at the maximum CH; and *a*, the rate of CH decrease in the late growth phase, in addition to *K*, the maximum saturation value; *d*_0_, the time point at the highest growth rate; and *r*, the growth rate. Our model proved suitable for predicting CL, DTH, SLW, and ADW. The highly heritable CH-related parameters *d*_1_ and *K* contributed to the prediction of DTH and CL. Notably, *d*_1_ also contributed to the prediction of SLW and ADW, possibly reflecting the association of biomass with duration of vegetative growth. The cultivar effects of traits measured by hand (CL, DTH, SLW, and ADW) and their corresponding CH-related parameters were highly correlated. These results indicate that CH-related parameters are useful for the prediction of traits usually measured by hand, reinforcing the significance of time-series monitoring by UAV in high-throughput phenotyping.

Desai et al. (2019) proposed a method to precisely estimate heading date by detecting flowering panicles in RGB images taken with a fixed camera every 5 min. Their method has the advantage of directly detecting panicles but is unsuitable for use by UAV because it requires a higher shooting frequency and a lower shooting altitude. On the other hand, our UAV method enabled us to predict DTH by focusing on the features of time-series CH changes in images taken weekly. Similarly, Zhao et al. (2021) proposed a method to predict wheat heading date by applying a logistic curve to growth data obtained by UAV, extracting the date when the second derivative is minimum. Our and their studies indicate that time-series models derived from UAV data can reveal developmental changes in crops in the field.

Our approach relies on applying a time-series model to CH data spanning crop growth from the vegetative stage to the reproductive stage in the field, and uses CH-related parameters as summary statistics of each trajectory. Time-series or longitudinal trait data have been modeled in several ways, including random regression with the Legendre polynomial. Although this polynomial can be incorporated into the expectation-maximization algorithm (Yang et al., 2006) and kernel methods (Campbell et al., 2018; Campbell et al., 2019), it is difficult to interpret the coefficients in the models. The coefficients of our CH-related parameters, on the other hand, have explicit meaning in the context of phenology and allow better interpretability.

The LME models decomposed each CH-related parameter into cultivar, year, and cultivar × year interaction effects. We considered year effect as an environmental effect and examined the influence of accumulated daily mean temperature on CH data. Our results indicate that *d*_0_, a CH-related parameter in the vegetative stage, is sensitive to the accumulated daily mean temperature, but *d*_1_, in the reproductive stage, is not. It is possible that DTH, associated with *d*_1_, is regulated by daylength, but growth is affected by temperature. In terms of cultivar effect, we showed strong correlations between *K* and CL, and between *d*_1_ and DTH, ADW, or SLW, suggesting the high contribution of these CH-related parameters to the prediction of each trait. This analysis can be useful in cultivar characterization. For example, in the case of cultivars “HKR” and “TYM”, the BLUPs of ADW with *d*_1_ deviated from linear (**Figure 7C**), probably reflecting their high yield and biomass.

CV by using cor and RMSE evaluated the robustness of the regression models to predict CL, DTH, ADW, GW, and SLW in an untested year and in untested cultivars. CV using cor estimates the magnitude of the correlation. In predicting CL, DTH, ADW, and SLW, values of cor by both CV methods were high. CV using RMSEs, which estimates the accuracy at predicting exact values of test data, can evaluate model robustness from the viewpoint of model variance, the phenomenon by which the prediction fluctuates with the training data, which results in variance of the predicted values (Bishop, 2006; Hastie et al., 2009). In predicting CL and DTH, RMSEs were similar by both types of CV methods. However, in predicting ADW and SLW, RMSEs of CV by year were about 1.5 times higher than those of CV by cultivar. Therefore, the prediction of ADW and SLW had model variance derived from year.

This study provides a novel method to predict traits that would usually be measured by hand from CH-related parameters extracted from aerial time-series data. The parameters did not prove useful in the prediction of GW, which manually measured data showed was not heritable. This indicates that GW is more sensitive to environment, suggesting the necessity of environmental data for the prediction of GW. We will examine new models for GW prediction from environmental data and other UAV-derived time-series data.

## Data availability statement

The raw data supporting the conclusions of this article will be made available by the authors, without undue reservation.

## Author contributions

ST, J-iY, and DO conceptualized the research; ST, TS, RI, YN, HT, AG, KM, SO, HM, YT, and TI performed the investigations; ST and TS developed the methodology; YN, AG, KM, SO, HM, YT, and TI provided the resources; ST, TS, RI, and YN curated data; HM, YT, TI, J-iY, and DO helped with funding acquisition; ST and DO wrote the manuscript; TS and JY reviewed and edited the manuscript. All authors contributed to the article and approved the submitted version.

## Supplementary material

Supplementary Figure 1 Trajectory of each CH time-series curve. The LME model calculated the cultivar effects of each CH-related parameter, which generated the cultivar-specific time-series curve (“Cultivar E”).

Supplementary Figure 2 Correlation plot of parameters and traits in **(A)** 2020 and **(B)** 2021. Values are correlation coefficients (cor); circles present them by color and size.

Supplementary Figure 3 Visualization of the LME model for GW. Left, proportion of 4 variance components; right top, BLUPs of cultivar and cultivar × year interactions of 30 cultivars; right bottom, BLUPs of 3 years. Cultivars are sorted by *d*_1_.

Supplementary Figure 4 Visualization of the LME model for ADW. Left, proportion of 4 variance components; right top, BLUPs of cultivar and cultivar × year interactions of 30 cultivars; right bottom, BLUPs of 3 years. Cultivars are sorted by *d*_1_.

Supplementary Figure 5 Visualization of the LME model for SLW. Left, proportion of 4 variance components; right top, BLUPs of cultivar and cultivar × year interactions of 30 cultivars; right bottom, BLUPs of 3 years. Cultivars are sorted by *d*_1_.

Supplementary Figure 6 Visualization of the linear mixed effects model for **(A)** *d*_1_ and **(B)** *d*_1_ *^temp^*. Left, proportion of 4 variance components; right top, BLUPs of cultivar and cultivar × year interactions of 30 cultivars; right bottom, BLUPs of 3 years. Cultivars are sorted by *d*_1_.
